# Suitable reference genes for real-time PCR in human HBV-related hepatocellular carcinoma with different clinical prognoses

**DOI:** 10.1186/1471-2407-9-49

**Published:** 2009-02-06

**Authors:** Li-Yun Fu, Hu-Liang Jia, Qiong-Zhu Dong, Jin-Cai Wu, Yue Zhao, Hai-Jun Zhou, Ning Ren, Qin-Hai Ye, Lun-Xiu Qin

**Affiliations:** 1 Liver Cancer Institute and Zhongshan Hospital, Fudan University, Shanghai, PR China; 2 Research Center for Cancer, Institutes of Biomedical Sciences, Fudan University, Shanghai, PR China

## Abstract

**Background:**

Housekeeping genes are routinely used as endogenous references to account for experimental differences in gene expression assays. However, recent reports show that they could be de-regulated in different diseases, model animals, or even under varied experimental conditions, which may lead to unreliable results and consequently misinterpretations. This study focused on the selection of suitable reference genes for quantitative PCR in human hepatitis B virus (HBV)-related hepatocellular carcinoma (HCC) with different clinical outcomes.

**Methods:**

We evaluated 6 commonly used housekeeping genes' expression levels in 108 HBV-related HCCs' matched tumor and non-tomor tissue samples with different clinical outcomes and 26 normal liver specimens by real-time PCR. The expression stability of the 6 genes was compared using the software programs geNorm and NormFinder. To show the impact of reference genes on data analysis, we took PGK1 as a target gene normalized by each reference gene, and performed one-way ANOVA and the equivalence test.

**Results:**

With the geNorm and NormFinder software programs, analysis of TBP and HPRT1 showed the best stability in all tissue samples, while 18s and ACTB were less stable. When 18s or ACTB was used for normalization, no significant difference of PGK1 expression (p > 0.05) was found among HCC tissues with and without metastasis, and normal liver specimens; however, dramatically differences (p < 0.001) were observed when either TBP or the combination of TBP and HPRT1 were selected as reference genes.

**Conclusion:**

TBP and HPRT1 are the most reliable reference genes for q-PCR normalization in HBV-related HCC specimens. However, the well-used ACTB and 18S are not suitable, which actually lead to the misinterpretation of the results in gene expression analysis.

## Background

With the application of quantitative real-time polymerase chain reaction (qPCR) in the high throughput and accurate expression profiling of selected genes, gene expression analysis is increasingly significant in many fields of biological research [1-3]. Nowadays, housekeeping genes (HKGs) are routinely-used as references in qPCR to normalize experimental data, such as differences in RNA quantity and quality, the overall transcriptional activity and differences in the cDNA synthesis [4], because, theoretically, HKGs are supposed to exhibit consistent, non-regulated, stable expression among different space-time and different tissues, even intervention models [5,6].

However, cancer development is a very complex stepwise process involving altered cell functions at many steps, through changing almost all genes in gene expression [7,8]. And many experimental evidences indicate that even the so-called HKGs are involved in tumorigenesis, including breast, prostate, colorectal, and bladder-cancer [9-16]. Typical HKGs including glyceraldehydes 3-phosphate dehydrogenase (GAPDH), beta-actin (ACTB), TATA-binding protein (TBP), 18S ribosomal RNA (18S) and many more have often been adopted from the literatures as reference genes without taking into account their specific tissue dependent behavior or the special design of the respective study [6,9-16]. Being de-regulated in various samples actually, those so-called HKGs for qPCR normalization on cancer research may lead to unreliable results and consequently misinterpretation [13,15,17]. Therefore, it is crucial to find appropriate reference genes for qPCR normalization on specific cases.

The major risk factor for the development of HCC is cirrhosis of the liver after chronic hepatitis virus infection. Recently, the geographical variability in the incidence of HCC has been attributed to the changing distribution and the natural history of hepatitis B virus (HBV) and hepatitis C virus (HCV) infection [18]. Therefore, HCV is the most important risk factor for HCC in western European and North American countries, while HBV is the major risk factor in East Asia, a distinct HCC subtype with an increasingly worldwide prevalence. However, evidence shows that HKG expression profile of HBV is distinct from HCV and relevant to hepatocarcinogenesis [19]. Recently, it was reported that in HCV-induced HCC, the combination of RPL41 and SFRS4 were the best to normalize qPCR data in USA [20], and there was no significant different in HKGs expression in the liver cancer tissues derived from HBV-infected and non-infected patients [21].

Based on one of the tumorigenesis and metastasis theories that genes favoring metastasis progression are initiated in the primary tumors [22,23], it is becoming a routine strategy to compare gene expression levels in tumor samples with different prognostic outcomes: cancer with- and without- metastasis [24-27], to find clinical prognosis biomarkers. Up to date, initial evidence shows GAPDH and ACTB are de-regulated in various TNM stages and tumor invasiveness in HCC [21]. Therefore, it is necessary to identify suitable reference genes relevant to HBV-related HCC with different clinical outcomes, which there is no previous systematic investigation yet.

This study focused on the commonly used HKGs as reference genes for q-PCR normalization in matched tumor and non-tumor tissue samples with different outcomes (with or without metastasis in 3 years following up) of HBV-related HCC and normal liver specimens. To select the commonly-used HKGs in HBV-related HCC, we searched on PubMed using the MeSH terms "hepatocellular carcinoma", "gene expression", and "RT-PCR" combined with the Boolean operator "AND" from January 2005 to March 2008 [15,28]. We evaluated 69 articles that had used various reference genes, and found that beta-actin (ACTB; 25 times; 36%), glyceraldehydes-3-phosphate dehydrogenase (GAPDH; 19 times; 28%), 18S-r RNA (18S; 12 times; 17%), TATA box binding protein (TBP; 5 times; 7%) and Hypoxanthine phosphoribosyl-transferase I (HPRT1; 4 times; 6%) and ribosomal protein L 13a (RPL13A; 4 times; 6%) were commonly used (Table 1). The six HKGs were selected, and their expression levels in normal liver tissues, tumor tissues (with-metastasis or without-metastasis HCC) and paired adjacent non-tumor liver tissues were compared to identify suitable reference genes for the purpose of normalization in HBV-related HCC.

**Table 1:** Six housekeeping genes evaluated in this study

Gene symbol	Gene name	Accession number	Primer/probe*
18S rRNA	18S ribosomal RNA	X03205	Hs99999901_s1

ACTB	β- Actin	NM_001101	Hs99999903_m1

GAPDH	Glyceraldehyde-3-phosphate dehydrogenase	NM_002046	Hs01922876_u1

HPRT1	Hypoxanthine phosphoribosyl-transferase I	NM_000194	Hs99999909_m1

RPL13A	Ribosomal protein, large,13A	NM_012423	Hs03043887_gH

TBP	TATA box binding protein	NM_003194	Hs00427620_m1

*ABI Gene expression Assay ID

## Methods

### Patients and specimens

Surgical tissue specimens from Chinese patients with primary HBV-related HCC were collected with informed consent and approved by the Institutional Review Board of the Liver Cancer Institute and Zhongshan Hospital, Fudan University (Shanghai, China). A total of 108 paired HCC tissues samples and adjacent non-malignant liver tissues were collected from the patients undergoing surgery at the Liver Cancer Institute during the period October 2003 to March 2005. The 108 paired samples were divided into with-metastasis and without-metastasis two subgroups based on their clinical prognostic features in 3 years following-up investigations. The clinicopathological characteristics of patients were presented in Table 2. All samples were histopathologically diagnosed as HCC according to Edmondson's classification. Pathologic diagnosis was independently done by two pathologists. An additional 26 normal liver specimens from patients with non-HCC liver disease were previously described [22]. The samples were sectioned immediately after surgical removal. Suitable tissue pieces were snap-frozen in liquid nitrogen and stored at -80°C until further processing.

**Table 2:** Clinic pathological characteristics of patients enrolled in this study

	HCC without metastasis (N = 63)		HCC with metastasis (N = 45)		Normal (N = 26)	
	
Clinical variable	n	%	n	%	n	%
Sex						

Male	46	73.02%	39	86.67%	9	34.62%

Female	17	26.98%	6	13.33%	17	65.38%

Age (years)						

≤ 50	33	52.38%	24	53.33%	16	61.54%

>50	30	47.62%	21	46.67%	10	38.46%

HBV						

+	63	100.00%	45	100.00%	26	100.00%

-						

Liver cirrhosis						

Yes	16	25.40%	7	15.56%	24	92.31%

No	47	74.60%	37	82.22%	2	7.69%

Unknown			1	2.22%		

Child-Pugh staging						

A	40	63.49%	36	80.00%	N/A	N/A

B	19	30.16%	9	20.00%	N/A	N/A

C	4	6.35%			N/A	N/A

Tumor size (cm)						

≤ 3	20	31.75%	27	60.00%	N/A	N/A

3~5	27	42.85%	18	40.00%	N/A	N/A

>5	16	25.40%			N/A	N/A

Edmondson grade						

I	3	4.76%	2	4.44%	N/A	N/A

II	50	79.37%	30	66.67%	N/A	N/A

III	8	12.70%	9	20.00%	N/A	N/A

Unknown	2	3.17%	4	8.89%	N/A	N/A

AFP (ng/ml)						

≤ 20	27	42.86%	14	31.11%	25	96.15%

20~200	11	17.46%	13	28.89%	1	3.85%

>200	25	39.68%	18	40.00%		

Abbreviation: N/A, not available

### RNA isolation and characterization

The tissue specimen were ground in liquid nitrogen and homogenized in Trizol (Invitrogen, Carlsbad, CA) using a poltroon homogenizer. Total RNA was purified following the RNeasy Mini protocol (Qiagen, Valencia, CA), including a DNaseI digestion, to avoid contamination with genomic DNA. The concentration of the isolated RNA and the ratio of absorbance at 260 nm to 280 nm (A260/A280 ratio) were measured with NanoDrop ND-1000 spectrophotometer (NanoDrop Technologies, Montchanin, DE, USA). The accuracy of the measurements on the NanoDrop spectrophotometer was previously controlled by comparative measurements using the conventional UV spectrophotometer Ultrospec 3000 (Pharmacia). The integrity of RNA was assessed with the RNA 6000 Nano LabChip kit using the Agilent 2100 Bioanalyzer (Agilent Technologies, Palo Alto, CA, USA). The electropherograms and gel-like images were evaluated with the Agilent 2100 Expert software that generates the RNA integrity number (RIN) to characterize RNA integrity. This number describes a gradual scale of RNA integrity from 1 (RNA completely degraded) to 10 (RNA without degradation), taking into account not only the conventional ratio of 28S to 18S ribosomal RNA (rRNA) but also other critical regions of the entire RNA electropherogram. The threshold inclusion values for the RNA samples were >1.90 for the A260/A280 ratio and >7 for the RIN value.

### First-strand cDNA synthesis

First-strand cDNA was synthesized using oligo dT and SuperScript™ III reverse transcriptase according to the manufacturer's instructions (Invitrogen, Carlsbad, CA, USA). Before transcription, RNA was denatured for 5 min at 65°C followed by cooling on ice. Finally, the Reverse Transcriptase was inactivated by heating the reaction mixture for 5 min at 93°C. All cDNAs was stored at -20°C until RT-PCR analysis. They were diluted 1:50 and 4.5 μl were used as template in a 10 μl qPCR reaction.

### Real-time PCR

The ABI Prism 7900 HT Sequence Detection System (Applied Biosystems, Foster City, CA, USA) was used for real-time PCR instruments. Essential gene-specific data are given in Table 1. The measurements on the ABI Prism System were performed with primers and TaqMan MGB probes as previously described [5]. The TaqMan probes were 5'-labeled with the reporter fluorescent dye 6-carboxy-fluorescein (FAM) and carried the quencher dye 6-carboxy-tetramethyl-rhodamine (TAMRA) labelled on a thymidine base near the 3'terminus. The amplification procedures were performed under the same reaction conditions as previously described in detail [5]. Briefly, the cycle conditions were set as follows: start with 2 min, 50°C step is required for optimal AmpErase^® ^UNG activity, 10 min template denaturation at 95°C, 40 cycles of denaturation at 95°C for 15 s, and combined primer annealing/elongation at 60°C for 1 min.

### Data analysis

Statistical analyses were performed with SPSS 15.0 for Windows (SPSS Software, Chicago, IL, USA). P values <0.05 were considered statistically significant. For stability comparison of candidate reference genes, we applied the software geNorm, version 3.4 [4], and NormFinder [29]. The program geNorm is a Visual Basic application tool for Microsoft Excel and is available on the Internet upon request by the programmers. CT values were converted into relative quantities for analysis with geNorm [4]. The program selects from a panel of candidate reference genes the two most stable genes or a combination of multiple stable genes for normalization. The NormFinder is also freely available on the Internet http://www.mdl.dk. It is a Microsoft Excel add-in and calculates the stability values of the individual candidate reference genes for normalization [29]. The stability value is based on the combined estimate of intra- and inter-group expression variations of the genes studied. A low stability value indicating a low combined intra- and inter-group variation proves high expression stability. Using this approach, the most stable single gene is calculated and an additional combination of two genes is recommended because the stability value of that combination is generally lower than that of the single gene.

## Results

### Expression levels of the "housekeeping" genes inHBV-related HCC

**Figure 1:**
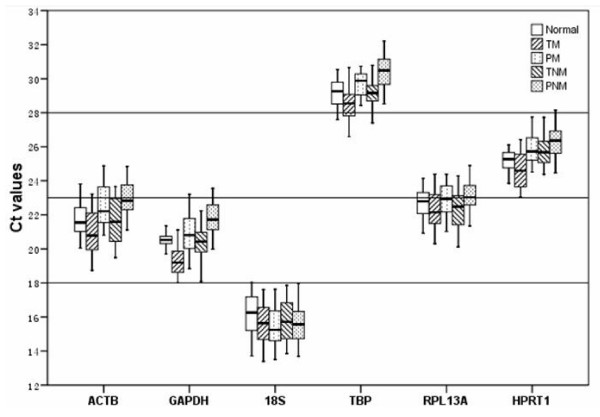
**Expression level of candidate reference genes**. We observed the six housekeeping genes in normal liver specimens(Normal), paired tumor and non-tumor liver specimens of HCCs with-metastasis (TM and PM), paired tumor and non-tumor liver specimens of HCCs without-metastasis (TNM and PNM). Values are given as real-time PCR cycle threshold numbers (Ct values). Boxes represent the lower and upper quartiles with medians. The arbitrary lines at Ct 18, 23 and 28 distinguish the groups of differently expressed housekeeping genes.

We observed the expression levels of the selected 6 housekeeping genes in different kinds of tissues, including normal liver tissue, malignant and paired non-malignant tissues from patients with HBV-related HCC. Their clinicopathological characteristics were shown in Table 2. The 6 housekeeping genes studied displayed a wide expression range, with the Ct values from 14 to 33 (Fig. 1). All of them showed an approximately normal distribution pattern proved by the Kolomogorov-Simirnov One-sample Test in all kinds of tissues tested. The expression levels were divided into three arbitrary ranges. The highest expression level with the lowest Ct value below 18 cycles was found in 18S; and a relatively lower expression level with the highest Ct value above 28 cycles was observed in TBP gene. The expression levels of GAPDH, ACTB, RPL13A and HPRT1 were higher than that of TBP with relatively lower Ct values ranged from 18 to 28 cycles.

### Expression stability of the housekeeping genes

**Figure 2:**
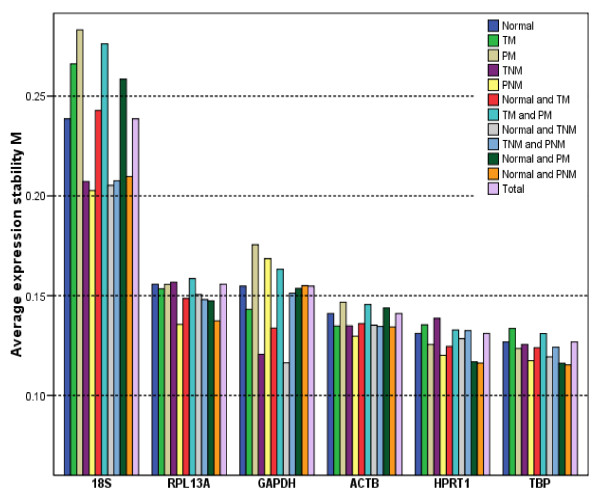
**Selection of the most suitable reference genes using geNorm analysis**. We performed geNorm analysis in variable combinations of each group according to different specific interest in cancer studies. The value of M was calculated for each gene, clustered in one group. The lowest M value characterizes genes with the most stable expression. The X-axis from left to right indicates the ranking of the genes according to their expression stability.

In search of the most stable reference genes, the expression stabilities of the tested genes were validated with two software programs, geNorm and NormFinder [4,29,30]. In the program geNorm, the expression stability of one gene was validated by calculating M value based on the average pairwise variation between all tested genes. The lowest M value characterizes the genes with the best stability. According to the published articles [4,30], stable genes' M values were below the default limit of 1.5 in the geNorm program. The average expression M values of the 6 candidate reference genes were demonstrated in Fig 2. The expression stabilities of the tested genes were different, with M values ranged from 0.1 to 0.3. The 18S was the least stable housekeeping gene with the M value of 0.239; TBP and HPRT1 were identified as the two most stable genes, with the M values of 0.127 and 0.131, respectively. In addition, we, for the first time, found the variance of the M value of these reference genes in different kinds of tissues (Fig. 2). Again, the M value of 18S was the most fluctuant gene among the six investigated HKGs, while TBP and HPRT1 were the most stable ones in all groups except the malignant without-metastasis (TNM) and the combination of the normal and TNM groups, in which GAPDH ranked the top.

**Figure 3:**
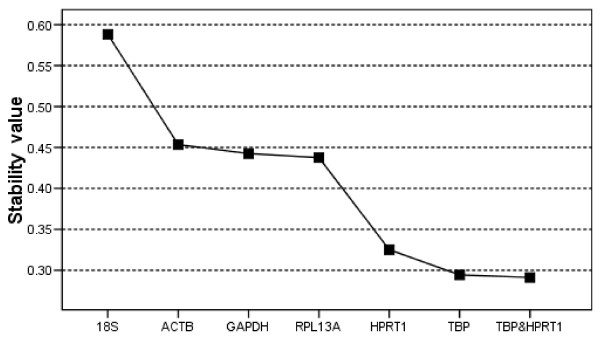
**Suitable reference genes for normalization and the best combination calculated by NormFinder program**. High expression stability is indicated by a low stability value as an estimate of the intra- and inter-group variation of the individual gene. The X-axis from left to right indicates the ranking of the genes according to their stability values.

The NormFinder program was also used to calculate the expression stabilities of the 6 reference genes, in which higher expression stability is indicated by a lower stability value as an estimate of the combined intra- and inter-group variation of the individual gene [29]. TBP and HPRT1 were still found to be the most stable genes, and TBP was the best one with a stability value of 0.294 (Fig 3). The combination (calculated geometric average [6]) of TBP and HPRT1 could improve the stability value to 0.291; however, there was not significant difference compared the stability of the combination with that of TBP alone (0.294).

### Significance of suitable reference gene's normalization forqPCR

**Figure 4:**
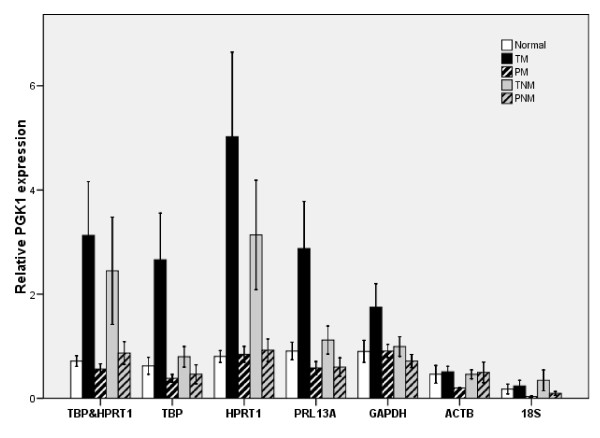
**Effect of different normalization approaches on the expression analysis of PGK1**. RT-PCR measurements of PGK1 and the corresponding reference genes were performed in 10 normal liver specimens (Normal), 11 pairs malignant (TM) and non-malignant (PM) with metastasis, 11 pairs malignant (TNM) and non-malignant (PNM) without metastasis liver samples. The Y-axis indicates the mean fold changes.

Phosphoglycerate kinase 1 (PGK1) plays an important role in tumour angiogenesis as a disulphide reductase. The secretion of PGK1 is regulated independently and inversely of its production and is consistent with the correlation between tumour hypoxia and angiogenesis [31,32]. To demonstrate the significance of suitable reference genes for normalization in order to get correct profiling data, we measured mRNA expression level of PGK1 in 10 normal liver tissues and 22 paired (tumor and non-tumor) tissue samples from patients with HCC (11 with-metastasis, 11 without-metastasis). The normalization of PGK1 expression was performed using different strategies: approaches with two reference genes (TBP and HPRT1) calculated by NormFinder and geNorm, or single (18S, RPL13A, ACTB, GAPDH, HPRT1, TBP) reference gene. The effect of different normalization approaches on the expression levels of PGK1 in different tissue samples were shown in Fig. 4. In addition, the P-values of the normalized PGK1 expression levels among the 5 sample groups were calculated (Table 3). When 18S and ACTB were used for normalization, no significant difference in the resulting relative gene expression levels of PGK1 (p > 0.05) could be found between the tumor with metastasis group (TM) and tumor without metastasis group (TNM) or normal liver tissues group (Normal). In contrast, when TBP, or the combination of TBP and HPRT1 were used, dramatic differences were found among the three groups (p < 0.001).

**Table 3:** Alterations (P values) in gene expression level of PGK1 by different reference genes' normalizations

Reference gene	Total^a^	Normal/TM^a^	Normal/TNM^a^	TM/TNM^b^
TBP and HPRT1	0.005	0.002	0.338	0.027

TBP	0.030	0.017	0.868	0.024

HPRT1	0.002	0.001	0.263	0.016

RPL13A	0.004	0.004	0.748	0.008

GAPDH	0.054	0.024	0.806	0.043

18S	0.343	0.717	0.312	0.504

ACTB	0.385	0.792	0.995	0.782

a: One-Way ANOVA

b: Paired-samples t test

## Discussion

In this study, we focused on qPCR data normalization in HBV-related HCC with different prognostic outcomes, where no conclusive systematic study comparing the suitability of different candidate reference genes has been published to date. All the experimental data and the results arising from the subsequent calculations were based on the particular design of the study: (1) using normal liver tissues for control; (2) dividing the HCCs into two groups, with-metastasis and without-metastasis according to 3 years following up; (3) enrolling modest samples to follow consequently statistical tests; (4) strictly controlling the isolated RNA; (5) using two different software programs to assess the candidate genes regarding their suitability as references; and (6) taking a target gene as an example to show the significance of suitable reference genes on normalization. All these characteristics of experimental design were significantly necessary for the reliability of data and the conclusions derived from them.

In the present study, the best-performing or combinations of reference genes were determined using the software programs geNorm as well as NormFinder [4,30]. Identifying suitable housekeeping genes is a both time consuming and expensive process, and has resulted in development of various programs and methods. The geNorm and NormFinder programs have been used in many studies to find suitable reference genes from a set of candidate genes [17,29,33]. In this study, 6 housekeeping genes, ACTB, GAPDH, 18S, HPRT1, RPL13A and TBP, were selected to evaluate their suitability as reference genes for relative quantification of target genes expression in HBV-related HCC. Both programs equally identified TBP and HPRT1 as the most stable combined reference genes. And more, TBP alone was shown to be stable enough as reference gene in this study. Recently, TBP is also recommended in the study of human renal cell carcinoma (RCC), although it is not as well-used as the other five references [28]. In our study, the use of TBP alone as control gene show sufficient (Fig. 3), which might because that our optimal study conditions resulting in high-quality RNA samples made only one reference gene necessary. HPRT1 was recommended as a universal, single reference gene for differential expression studies in cancer research [34]. However, based on the results of this study, it seemed to be not as stable as TBP, at least in HBV-related HCC. For the gene expression study in HCC tissues, appropriate reference genes in a range similar to the target genes are recommended for normalization.

In this study, our findings indicate that the use of inappropriate genes for normalization can lead to under- or over-estimations of the relative gene expression levels or to misinterpretations of the results. The normalization for target genes expression was used as an example to illustrate the essentiality of a suitable reference gene selected from a panel of candidate reference genes. We used PGK1, which plays a significance role in HCC tumourigenesis [31,32], as a target gene. The results showed that unsuitable reference genes led serious gene quantification error interpretations. We recommend the use of TBP and HPRT1 for normalizing expression results using the geometric averaging of the two reference genes [4]. Although the advantage of using both reference genes for normalizing is not clearly evident than using the most stable gene TBP alone in our study, a more accurate normalization was found by other investigators when more than one reference gene was used [4,33,35].

In addition, all HCC samples in this study were obtained from HBV-positive Chinese patients. It remains to be determined whether this result also can be applied to other populations. Recently, it was reported that in HCV-induced HCC the combination of RPL41 and SFRS4 were best to normalize qPCR data in USA [20]. Studies are under way to further explain why those housekeeping genes are deregulated in different conditions, and more molecular mechanism waits to find out.

## Conclusion

In conclusion, comparing gene expression levels in moderate specimens, including normal liver tissues, paired tumor and non-tumor tissues of patients with HCC (including different status of metastasis), our research revealed two appropriate genes TBP and HPRT1 were reliable for normalization in the study of HBV-related HCC. In addition, the effects of differentially expressed genes in qPCR normalization were presented. Some of the "housekeeping genes", such as 18S and ACTB, were found lead to misinterpretations on experimental data because of their unstable gene expressions in those tissues mentioned above.

## Abbreviations

HBV: Hepatitis B virus; HCC: Hepatocellular carcinoma; qPCR: Quantitative polymerase chain reaction; HKGs: Housekeeping genes; HCV: Hepatitis C virus.

## Competing interests

The authors declare that they have no competing interests.

## Authors' contributions

LYF carried out the study, and drafted the manuscript. HLJ, QHY, NR, and HJZ participated in the design of this study. JCW assisted in the statistical analysis. QZD and YZ participated in correcting patient sample's following-up investigation data. LXQ supervised this study, and involved in revising it critically for important intellectual content. All authors read and approved the final manuscript.

## Pre-publication history

The pre-publication history for this paper can be accessed here:

http://www.biomedcentral.com/1471-2407/9/49/prepub

## References

[B1] Heid CA Stevens J Livak KJ Williams PM Real time quantitative PCR Genome Res 1996 6 986 994 10.1101/gr.6.10.986 8908518

[B2] Bustin SA Absolute quantification of mRNA using real-time reverse transcription polymerase chain reaction assays J Mol Endocrinol 2000 25 169 193 10.1677/jme.0.0250169 11013345

[B3] Rasmussen R Meuer S, Wittwer C, Nakagawara K Quantification on the LightCycler Rapid Cycle Real-time PCR. Methods and Applications 2001 Heidelberg: Springer Press 21 34

[B4] Thellin O Zorzi W Lakaye B De BB Coumans B Hennen G Grisar T Lgout A Heinen E Housekeeping genes as internal standards: use and limits J Biotechnol 1999 75 291 295 10.1016/S0168-1656(99)00163-7 10617337

[B5] Radonic A Thulke S Mackay IM Landt O Siegert W Nitsche A Guideline to reference gene selection for quantitative real-time PCR Biochem Biophys Res Commun 2004 313 856 862 10.1016/j.bbrc.2003.11.177 14706621

[B6] Vandesompele J De Preter K Pattyn F Poppe B Van Roy N De Paepe A Speleman F Accurate normalization of real-time quantitative RT-PCR data by geometric averaging of multiple internal control genes Genome Biol 2002 3 7 research0034.1 research0034.11 10.1186/gb-2002-3-7-research0034 PMC126239 12184808

[B7] Brumy AM Richardson HE Using Drosophila melanogaster to map human cancer pathways Nat Rev Cancer 2005 5 8 626 639 10.1038/nrc1671 16034367

[B8] Kopper L Timar J Genomics of prostate cancer: is there anything to "translate"? Pathol Oncol Res 2005 11 4 197 203 10.1007/BF02893851 16388315

[B9] Lyng M Laenkholm A-V Pallisgaard N Ditzel H Identification of genes for normalization of real-time RT-PCR data in breast carcinomas BMC Cancer 2008 8 1 20 18211679 10.1186/1471-2407-8-20 PMC2248196

[B10] Hsiao LL Dangond F Yoshida T Hong R Jensen RV Misra J Dillon W Lee KF Clark KE Haverty P A compendium of gene expression in normal human tissues Physiol Genomics 2001 7 2 97 104 11773596 10.1152/physiolgenomics.00040.2001

[B11] Butte AJ Dzau VJ Glueck SB Further defining housekeeping, or "maintenance" genes focus on "A compendium of gene expression in normal human tissues" Physiol Genomics 2001 7 2 95 96 11773595 10.1152/physiolgenomics.2001.7.2.95

[B12] De Kok JB Roelofs RW Giesendorf BA Pennings JL Waas ET Feuth T Swinkels DW Span PN Normalization of gene expression measurements in tumour tissues: comparison of 13 endogenous control genes Lab Invest 2005 85 1 154 159 15543203 10.1038/labinvest.3700208

[B13] Schmittgen TD Zakrajsek BA Effect of experimental treatment on housekeeping gene expression: validation by real-time, quantitative RT-PCR J Biochem Biophys Methods 2000 46 69 81 10.1016/S0165-022X(00)00129-9 11086195

[B14] Goidin D Mamessier A Staquet MJ Schmitt D Berthier VO Ribosomal 18S RNA prevails over glyceraldehydes-3-phosphat dehydrogenate and beta-actin genes as internal standard for quantitative comparison of mRNA levels in invasive and non-invasive human melanoma cell subpopulations Anal Biochem 2001 295 17 21 10.1006/abio.2001.5171 11476540

[B15] Ohl F Jung M Xu C Stephan C Rabien A Burkhardt M Nitsche A Kristiansen G Loening SA Radonic A Gene expression studies in prostate cancer tissue: which reference gene should be selected for normalization? J Mol Med 2005 83 12 1014 1024 10.1007/s00109-005-0703-z 16211407

[B16] Khimani AH Mhashilkar AM Mikulskis A O'Malley M Liao J Golenko EE Mayer P Chada S Killian JB Lott ST Housekeeping genes in cancer: normalization of array data Biotechniques 2005 38 5 739 745 10.2144/05385ST04 15948292

[B17] Dheda K Huggett JF Chang JS Kim LU Bustin SA Johnson MA Rook GA Zumla A The implications of using an inappropriate reference gene for real-time reverse transcription PCR data normalization Anal Biochem 2005 344 141 143 10.1016/j.ab.2005.05.022 16054107

[B18] Liu CJ Kao JH Hepatitis B virus-related hepatocellular carcinoma: epidemiology and pathogenic role of viral factors J Chin Med Assoc 2007 70 141 145 17475593 10.1016/S1726-4901(09)70346-6

[B19] Pang R Tse E Poon RT Molecular pathways in hepatocellular carcinoma Cancer Lett 2006 240 157 169 10.1016/j.canlet.2005.08.031 16239065

[B20] Waxman S Wurmbach E De-regulation of common housekeeping genes in hepatocellular carcinoma BMC Genomics 2007 8 243 251 17640361 10.1186/1471-2164-8-243 PMC1937003

[B21] Gao Q Wang XY Fan J Qiu SJ Zhou J Shi YH Xiao YS Xu Y Huang XW Sun J Selection of reference genes for real-time PCR in human hepatocellular carcinoma tissues J Cancer Res Clin Oncol 2008 134 979 986 10.1007/s00432-008-0369-3 18317805 PMC12160763

[B22] Ye QH Qin LX Forgues M He P Kim JW Peng AC Simon R Predicting hepatitis B virus-positive metastatic hepatocellular carcinomas using gene expression profiling and supervised machine learning Nat Med 2003 9 416 423 10.1038/nm843 12640447

[B23] Budhu A Forgues M Ye QH Jia HL He P Zanetti KA Kammula US Chen YD Qin LX Tang ZY Wang XW Prediction of venous metastases, recurrence, and prognosis in hepatocellular carcinoma based on a unique immune response signature of the liver microenvironment Cancer Cell 2006 10 99 111 10.1016/j.ccr.2006.06.016 16904609

[B24] Qin LX Tang ZY Sham JS Ma ZC Ye SL Zhou XD Wu ZQ Trent JM Guan XY The association of chromosome 8p deletion and tumor metastasis in human hepatocellular carcinoma Cancer Res 1999 59 5662 5665 10582679

[B25] Khan J Wei JS Ringnér M Saal LH Ladanyi M Westermann F Berthold F Schwab M Antonescu CR Peterson C Meltzer PS Classification and diagnostic prediction of cancers using gene expression profiling and artificial neural networks Nat Med 2001 7 673 679 11385503 10.1038/89044 PMC1282521

[B26] Budhu A Forgues M Ye QH Jia HL He P Zanetti KA Kammula US Chen Y Qin LX Tang ZY Wang XW Prediction of venous metastases, recurrence, and prognosis in hepatocellular carcinoma based on a unique immune response signature of the liver microenvironment Cancer Cell 2006 10 99 111 10.1016/j.ccr.2006.06.016 16904609

[B27] Budhu A Jia HL Forgues M Liu CG Goldstein D Lam A Zanetti KA Ye QH Qin LX Croce CM Tang ZY Wang XW Identification of metastasis-related microRNAs in hepatocellular carcinoma Hepatology 2008 47 897 907 10.1002/hep.22160 18176954

[B28] Jung M Ramankulov A Roigas J Johannsen M Ringsdorf M Kristiansen G Jung K In search of suitable reference genes for gene expression studies of human renal cell carcinoma by real-time PCR BMC Mol Biol 2007 8 47 59 17559644 10.1186/1471-2199-8-47 PMC1913536

[B29] Andersen CL Jensen JL Orntoft TF Normalization of real-time quantitative reverse transcription-PCR data: a model-based variance estimation approach to identify genes suited fro normalization, applied to bladder and colon cancer data sets Cancer Res 2004 64 5245 5250 10.1158/0008-5472.CAN-04-0496 15289330

[B30] Vandesompele J De Preter K Pattyn F Poppe B Van Roy N De Paepe A Speleman F GeNorm software manual, update 6 Sep 2004 http://medgen.ugent.be/~jvdesomp/genorm

[B31] Lay AJ Jiang XM Kisker O Flynn E Underwood A Condron R Hogg PJ Phosphoglycerate kinase acts in tumor angiogenesis as a disulphide reductase Nature 2000 408 869 873 10.1038/35048596 11130727

[B32] Hwang TL Liang Y Chien KY Yu JS Overexpression and elevated serum levels of phosphoglycerate kinase 1 in pancreatic ductal adenocarcinoma Proteomics 2006 6 2259 2272 10.1002/pmic.200500345 16493704

[B33] Tricarico C Pinzani P Bianchi S Paglierani M Distante V Pazzagli M Bustin SA Orlando C Quantitative realtime reverse transcription polymerase chain reaction: normalization to rRNA or single housekeeping genes is inappropriate for human tissue biopsies Anal Biochem 2002 309 293 300 10.1016/S0003-2697(02)00311-1 12413463

[B34] Szabo A Perou CM Karaca M Perreard L Quackenbush JF Bernard PS Statistical modeling for selecting housekeeper genes Genome Biol 2004 5 R59 68 15287981 10.1186/gb-2004-5-8-r59 PMC507884

[B35] Schmid H Cohen CD Henger A Irrgang S Schlöndorff D Kretzler M Validation of endogenous controls for gene expression analysis in microdissected human renal biopsies Kidney Int 2003 64 356 360 10.1046/j.1523-1755.2003.00074.x 12787429

